# Supplementary material for: An exploration of multi-level factors affecting routine linkage to HIV care in Zambia’s PEPFAR-supported treatment program in the treat all era
Source: PLOS Glob Public Health. 2024 May 23;4(5):e0003094. doi: 10.1371/journal.pgph.0003094 (PMC11115257; doi:10.1371/journal.pgph.0003094)
Supplement: Attachment — Submitted filename: Response to Reviewers.docx [file pgph.0003094.s002.docx]

**Reviewer #1: The authors present the results of a qualitative study of facilitators and barriers to full linkage to ART from 10 facilities in Lusaka, Zambia. While the paper addresses an important topic and is well written, there are several areas the authors may wish to consider.**

**Minor comments:**
**1) please include information on the IRB or ethics committee that approved the study.**

Response: Thank you for your guidance. We have included this information on page 10, line 214 – 216 under ethics statement.

**2) It is unclear how the authors established "silent transfer" from the routine records. If the definition is transfer without notifying the first clinic it would imply that this was not in the records. Was silent transfer status established after contacting the potential participant?**

Response: Thank you for this comment. The first clinic or the clinic from which they left without notification will not have records of where they would have gone. However, the clinic to which they transfer will have previous ART treatment records which can be reviewed by querying the electronic medical record system for HIV care, SmartCare. This provides information that the participant may have ‘silent transferred’ from another clinic. From a list of clinic attendees who *may* have transferred silently, we then confirmed silent transfer by contacting potential participants during study recruitment. Thus, a ‘silent transfer’ participant was established through a two-step process of review of the electronic medical record in consultation with the clinic staff who had prior knowledge of potential silent transfers, and then, secondly, contacting potential participants to confirm.

We have added a line to make this clearer on page 5-6, line 110-115.

**3) figure 1 either doesn't need the single arrow from reasons to VCT or arrows from reasons need to be added going to PITC and CBT.**

Response: Thank you for your suggestion, we have made the necessary changes to the figure.

**4) no need to abbreviate if only used once (e.g. LMIC on line 437)**

Response: Thank you for noting this. We have expanded the use of the abbreviation LMIC in our revised manuscript in the introduction section and hence maintained the abbreviation.

**Major comments:**
**1) Table 2 would make more sense as row percentages rather than column percentages. If the authors are looking to interpret the characteristics that are associated with the linkage stage of the participant, some nuances are lost when looking within linkage stage rather than among those with a particular characteristic what is the distribution of linkage stage within that characteristic.**

Response: Thank you for this suggestion, we agree that row percentage will add more nuance to the findings. We have converted the Table to now reflect row percentages and have briefly mentioned the main observations from the Table in the Results text.

**2) while the quotes from the qualitative interviews are good, since the purpose of the paper is to identify barriers and facilitators of full linkage, it may be more helpful to also identify the linkage stage along with marital status, sex, and age in the reasons for testing section.**

Response: This is a great suggestion. We have added the linkage status after each of the two quotes in the reasons for testing section on line 256 and 270-271, respectively.

**3) in Table 2 you report 11 people were silent transfers but on line 356-357 you report 10 participants 8 women and 2 men rather than 3 men as shown in table 2.**

Response: Thank you for noting this typographical error. We have aligned these figures under the text in the silent transfer section to reflect the correct sample size presented in the table. Please see this change on line 428 & 429.

**4) Similarly comparing whole numbers of women to men in the results is not very informative. There are more women in your sample and there are more women living with HIV in Zambia than men. when discussing linkage stage, silent transfer for example, rather than saying 8 and 2 (or 8 and 3 as in table 2) those represent 21% of women and 15% of men. Presenting as frequencies ignores the overall sex imbalance, e.g. you would expect more women by chance alone.**

Response: We have noted your comment and reflected proportions where we are comparing numbers of women to men. Specifically, this has been reflected under the section No ART Pick-up (line 307 & 317) and Silent Transfer (line 429).

**Reviewer #2: Comment to authors**

**This is a very well-written manuscript. The 2016 data presented in the first two paragraphs about ART use are very old, authors need to provide the current data.
It is great that this study focuses on a very important aspect for people living with HIV. However, several aspects need to be addressed to improve the manuscript:**

**INTRODUCTION**

1. **The 2016 data presented in the first two paragraphs about ART use are very old, authors need to provide the current data.**

Response: We have updated the HIV epidemiology and ART use data in the first two paragraphs according to the reviewer’s suggestion.

2. **The main focus of this paper is on understating enablers and barriers to the linkage to HIV care, but there is no real synthesis of findings of the existing studies globally and in Zambia about (i) enablers/facilitators to the linkage to HIV care/service, and (ii) barriers to the linkage to HIV care/services.**

Response: We thank the reviewer for their careful review and for sharing important manuscripts from the literature on linkage to HIV care. We would like to clarify that we are abreast with the existing evidence on barriers and facilitators to linkage to care in routine HIV care settings and recognize that there is a long-standing global literature in this area, to which we have contributed (please see references below from our group that we cite in the paper):

1. *Pry, J., Chipungu, J., Smith, H.J., Moore, C.B., Mutale, J., Duran‐Frigola, M., Savory, T. and Herce, M.E., 2020.* ***Patient‐reported reasons for declining same‐day antiretroviral therapy initiation in routine HIV care settings in Lusaka, Zambia: results from a mixed‐effects regression analysis****.  J Int AIDS Soc*. 2020 Jul;23(7):e25560. doi: 10.1002/jia2.25560. PMID: 32618137*.*
2. *Herce ME, Chi BH, Liao RC, Hoffmann CJ.* ***Re-thinking Linkage to Care in the Era of Universal Test and Treat: Insights from Implementation and Behavioral Science for Achieving the Second 90****. AIDS and behavior. 2019;23(Suppl 2):120-8.*
3. Mody, A., Sikazwe, I., Namwase, A.S., Mwanza, M.W., Savory, T., Mwila, A., Mulenga, L., Herce, M.E., Mweebo, K., Somwe, P. and Eshun-Wilson, I., 2021. **Effects of implementing universal and rapid HIV treatment on initiation of antiretroviral therapy and retention in care in Zambia: a natural experiment using regression discontinuity**. The Lancet HIV, 8(12), pp.e755-e765.

While the linkage to care literature dates back over 20 years, in our study, we were specifically interested in re-examining these factors in the context of universal test-and-treat and same-day HIV treatment initiation in Zambia. In particular, we aimed to draw out factors along a series of steps from HIV testing to HIV care enrolment that frame linkage to care as a complex process even though treatment eligibility had been simplified. The data follow a conceptual model, tailored to the test-and-treat era, referenced in the paper mentioned above by Herce ME and colleagues published in *AIDS and Behavior*.

As we appreciate this point may not have come out clearly in the manuscript, we have added more detail in the Introduction highlighting known barriers to HIV care linkage and the unique knowledge gap that this research aimed to fill. We also more clearly emphasize throughout the Discussion section the implications are findings have for the existing HIV linkage literature and HIV programming in high burden settings in sub-Saharan Africa.

**3. This synthesis will help you to identify research/knowledge gap(s) in the literature, which you bridge through your study, and that becomes your contribution to knowledge. In other words, what is new (novelty) about your study? Why is it important to research this particular topic with this study participants?**

**I would suggest the authors go through the following studies and look at the references used to help you make the synthesis. This synthesis will be very helpful once you discuss the findings.**

Response: Thank you for pointing us to important publications in the existing literature which we have reviewed. We especially appreciate the publications that report on specific barriers such as stigma and other social influences among the general population as a barrier to HIV care as this aligns well with our findings.

Please note that our study was conducted in urban Lusaka, Zambia, in southern Africa, and was embedded within a routine PEPFAR-supported HIV treatment programme. While we added particularly relevant references provided by the reviewer, most of our literature review focused on studies from general populations living with HIV in sub-Saharan Africa. Literature focused on key populations, rural areas, or other regions globally may illuminate broadly relevant enabling and impeding factors for care linkage, but may lack contextual relevance to properly situate our finding within the broader HIV literature.

As noted above, we deliberately focused our study on experiences and factors new to linkage to care since the advent of test and treat policy in Zambia, and how these hinder or facilitate linkage to care. Hence, our research was nested in a routine test-and-treat programme where patients were offered opt-out HIV testing and HIV treatment if positive, all on the same day.

In the revised manuscript, we now highlight key factors influencing linkage to care identified in the important studies shared by the reviewer and more clearly indicate how these factors have evolved or remained the same in the test-and-treat era. We also more explicitly emphasize the novelty of our study in the first paragraph and throughout the Discussion section. Namely, we re-examine barriers and enablers to care linkage in the new context of universal test-and-treat and through the lens of a conceptual model that envisions linkage to care as its own nuanced pathway in the HIV care cascade. We explicitly point out in the Discussion how the current policy of test and treat may have exacerbated previously well-established barriers to linkage to care.

**METHODS
Who did the interviews. I would suggest the authors using COREQ checklist to help improve their reporting on the methods section:** [**https://www.equator-network.org/reporting-guidelines/coreq/**](https://www.equator-network.org/reporting-guidelines/coreq/) **Who did the transcription? Who are the 3 coders? Are the coders the interviewers? If they are different people, how did you manage the non-verbal language, gestures, etc that were observed during interviews? How did you solve discrepancies among the 3 coders during the coding process? Authors need to provide the thematic analysis steps used.**

Response: We would like to thank the Reviewer for suggesting using COREQ to organize the Methods section. We have adapted this section to reflect relevant headings and details suggested in the checklist.

A team of research assistants conducted the interviews, while a separate team of transcribers was employed to do the transcription. While we understand the benefits of having the data collectors transcribe the data, time was a limiting factor in allowing this to happen. Non-verbal language, gestures, e.t.c were noted down in a summary form that each research assistant had to complete after conducting the interviews and were reviewed by the coders as they familiarized themselves with the transcripts. The social and behavioural researcher conducted quality checks on a sample of transcripts by listening to the audio recordings while reading the transcriptions verbatim.

We have clarified our use of Braun and Clarkes thematic analysis six step process under the data analysis section.

**DISCUSSION
“Recurrent themes of internal and external stigma, concerns about medication side effects, and a perceived lack of partner and social support, appear to impede linkage to care at every step along the linkage pathway, while quality counseling, patient-centered health worker engagement, and family, peer, and other social network supports seemed to help overcome these barriers.”

These findings are not new and have been reported in previous studies. Please consult the following studies and the references they used:**

Response: In response to this and the previous comment from Reviewer #2 about novelty, we have revised the Discussion section to more clearly connect our study findings to the wider linkage literature, particularly from before the test-and-treat era, and also map the findings from our work that are new and build on previous work. To avoid confusion, we have removed the statement “for the first time to our knowledge” in the Discussion section.

**CONCLUSION
What are the implications of your findings for future studies and HIV policies and practice in the study setting?**

Response: We further detail the implications of our findings throughout the Discussion section. For example, we discuss the implications of “silent transfers” and the burden placed on the health system (line 570 – 580). In the last two paragraphs, we discuss strategies such as patient-centered HIV services to facilitate full linkage. We conclude with a statement on how the health system can be better organized to improve linkage to care.
